# Gene expression of beta carotene genes in transgenic biofortified cassava

**DOI:** 10.1007/s13205-014-0243-8

**Published:** 2014-08-28

**Authors:** P. K. Telengech, J. N. Maling’a, A. B. Nyende, S. T. Gichuki, B. W. Wanjala

**Affiliations:** 1 Institute for Biotechnology Research, Jomo Kenyatta University of Agriculture and Technology, PO Box 62000-00200, Nairobi, Kenya; 2 Kenya Agricultural Research Institute, Biotechnology, Biodiversity and Conservation Centre, PO Box 14733-00800, Nairobi, Kenya; 3 PO Box 45-30307, Mosoriot, Kenya

**Keywords:** Confined field trial, Beta carotene genes, Gene expression, Patatin promoter, RT-PCR

## Abstract

Cassava is an important food for millions of people around the world. However, cassava is deficient in protein, iron, zinc, pro-vitamin A and vitamin E. Cassava biofortified with pro-vitamin A can help reduce Vitamin A Deficiency among the undernourished communities that rely upon it for sustenance. BioCassava Plus project has developed transgenic cassava that expresses beta carotene in roots using root specific patatin promoter. This study aimed at confirming expression of *npt*II, *crtB* and DXS genes. Leaf and roots samples were obtained from confined field trial at KARI Alupe at 12 months after planting. RNA was isolated from cassava roots and leaves using a modified Dellaporta protocol, analyzed for expression of DXS*, crtB* and the selectable marker*, npt*II using the one step RT-PCR kit (Qiagen) and analyzed through gel electrophoresis. DXS, *crtB* and *npt*II genes were expressed in the roots as anticipated. On the contrary, DXS and *crtB* genes were also expressed in the leaves of the transgenic cassava despite the use of root specific patatin promoter. This finding indicates that the promoter confers expression in leaves too. Intensive molecular screening of the biofortified transgenic cassava is important for risk assessment that informs on integrity of the promoter gene and confirms expression of the beta carotene genes.

## Introduction

Cassava (*Manihot esculenta* crantz, Euphorbiaceae) is an important staple food crop. It accounts for more than 50 % of daily energy to more than 200 million people in sub-Saharan Africa (SSA) and about 700 million people worldwide (Manyong et al. 2004; HARVESTPLUS 2008). In Kenya, cassava is grown in Western, Eastern, Central parts of Rift Valley and Coastal regions where it accounts for 9 % of the total calories in the diet. It is a major root crop ranked second after Irish potato (Ministry of Agriculture Kenya 2007). It is consumed as raw, cooked, boiled, fried slices, flakes, fermented*, gari, fufu, konkonte* among others (Manyong et al. 2004; Kimathi et al. 2007; Obiero et al. 2007). In addition, some ethnic communities and regions are known to utilize cassava leaves as food and feed due to their nutritive value (Horna et al. 2006). Cassava leaves are good sources of protein, vitamins and minerals which lack in cassava roots (Lancaster and Brooks 1983). Consumption is higher in Coast, Central and Western regions in respective percentages 89.3, 40.9 and 22.4 % (Kariuki et al. 2002). In the Coastal region, more than 60 % of the farmers use the leaves as livestock feed (Kiura et al. 2005).

Nutrients like protein, vitamin A and E, iron, and zinc are low in roots leading to malnutrition in cassava growing and consuming regions of Kenya (Alison et al. 2010). Strategies addressing Vitamin A Deficiency (VAD) include food fortification and pharmaceutical supplementation; which are unsustainable to the resource poor communities (Angela 2007). Biotechnological approaches offer an alternative to food fortification as has been the case with golden rice 2–Vitamin A in Asia (Paine et al. 2005), mustard oil-herbicide tolerance in India and canola-herbicide tolerance in USA (Shewmaker et al. 1999). Efforts have been made to biofortify the cassava to increase nutrients such as vitamin A, protein and iron.

Proof of concept has been ongoing for accumulation of β carotene a precursor of Vitamin A in the model cultivar TMS 60444 in Puerto rico, Uganda and Kenya (Fregene et al. 2010). In Puerto Rico it was found out that the biofortified cassava grown in green house and CFT contains up to 40 µg/g dry weight (DW) of beta carotene (Vitamin A) (Fregene et al. 2010). The transgenic cassava was transformed with two transgenes: *crtB* phytoene Synthase gene from *Erwinia herbicolor* and 1-deoxy-d-xylulose-5-phosphate synthase gene (DXS) from *Arabidopsis thaliana.* The *crtB* gene drives the committed step in β carotene synthesis using geranylgeranyl-diphosphate (GGDP) in the plastid isoprenoid pathway. DXS is intended for increased concentration of GGDP for β-carotene synthesis. These two genes are under the control of Patatin promoter from *Solanum tuberosum* (Potato) (Fregene et al. 2010).

Gene expression is controlled at the level of transcription achieved by the promoter. There is need to understand the integrity of the patatin promoter; that is tuber specific in potato and transgenic cassava (Naumkina et al. 2007). On the contrary, though the patatin is tuber specific; it could also be induced by different endogenous and exogenous factors in the leaves such as sucrose, light and other factors in other plants. The genes encoding patatin are divided into two: Class I and Class II genes. The class I promoter is actively expressed in developing tubers whereas their expression in leaves could be induced by sucrose treatment (Rotcha-Sosa et al. 1989; Jefferson et al. 1990). The *beta glucoronidase* *(GUS*) gene under the control of class I patatin promoter in the transgenic potato is sufficient to drive tuber specific and sucrose inducible expression of the fused *GUS* gene. The class I patatin promoter was examined for expression in *Arabidopsis* plant and it was discovered that the promoter was tissue specific and induced by sucrose or proline in the leaves (Martin et al. 1997; Hellmann et al. 2000).

There is need to ensure that the transgenic cassava developed is safe and the genes expressed are root specific. The biofortified cassava inserted genes should be expressed in roots. Expression of the genes in other tissues calls for further risk analysis in those specific tissues such as transgenic cassava leaves which are utilized in other communities as food and feed. The present study sought to establish the expression of the DXS*, crtB* and *npt*II genes under control of patatin promoter. Reverse transcriptase PCR was utilized to ascertain the specificity of the promoter. RT-PCR can only inform of whether expression takes place or not (qualitative).

## Materials and methods

### Transgenic cassava

The transgenic cassavas were produced at the University of Nebraska, USA at the Ed Cahoon Lab in collaboration with Donald Danforth Plant Science Centre, St. Louis, USA (DDPSC) (Centre 2013). The transgenic cassavas were provided by DDPSC through KARI Biotechnology programme who were the custodians of the CFT. The events were produced through *Agrobacterium* mediated gene transformation cells of cassava cultivar TMS 60444 with DNA constructs carrying two codon optimized genes: phytoene Synthase (*crtB*) obtained from *Erwinia herbicolor* and DXS sequences gene from *Arabidopsis thaliana* encoded by CLA1 gene and *npt*II selectable marker gene (BC+ Dossier). The plasmid vector *pILTAB505* used to create the transgenic cassava is referred to as *pCAMBIA*. This vector is a standard *pCAMBIA* binary vector for plant transformation with *Agrobacterium tumefaciens.* Marker *npt*II was fused to a CaMV 35S promoter from Cauliflower Mosaic Virus used to drive the expression of *npt*II and to the 3′ UTR polyadenylation sequence for the nopaline synthase from *Agrobacterium tumefaciens.* The *Solanum tuberosum* patatin promoter was used to confer storage root expression of the *crtB* and DXS transgenes. The *crtB* and DXS transgenes were linked at their 3′ termini to the 3′ UTR polyadenylation sequence for *npt*II.

DDPSC provided four transgenic lines expressing the *crtB* and DXS genes and one wild type as control. The transgenic and wild type cassava were of the cultivar TMS 60444. A total of 100 healthy plantlets of four transgenic lines labeled DXS *crtB 2*, DXS *crtB 37,* DXS *crtB 73* and DXS *crtB 20* were imported. A total number of 250 wild type cassava plantlets were also imported from USA by KARI. The plants were transported to KARI Kakamega Screen house for 8 weeks to undergo acclimatization and hardening. This was achieved through controlling temperature and relative humidity, lighting and irrigation (Ospina et al. 2007). Standard operating procedures for transport and storage were observed at every stage of transportation as defined in the Cassava CFT Handbook (UNCST 2009). In addition to the imported varieties, a local naturally yellow fleshed variety containing total carotenoid (*Nyaboda*) was obtained from KARI Kakamega as a second control and served as a local check. A total of 25 clean stakes were collected from KARI Kakamega.

### Experiment site: confined field trial

The plantlets were transported from Kakamega screen house in a three tier system (primary, secondary and tertiary packaging) to the CFT site at Kenya Agricultural Research Institute (KARI) Alupe, 8 km from Busia Town in Teso District, Western Province for experimentation. The coordinates of site are: 0°29.9′ N 34°7.5′ E; elevation 1,181 m above sea level. The soil is deep sandy loam. The average rainfall is 980 mm while the temperature is 24.8 °C (Jaetzold et al. 2009).

Mitigation measures to prevent gene introgression and persistence in environment were achieved as required by the Kenyan law (Biosafety Act 2009).

### Experimental layout

The experiment was planted in randomized complete block design. It consisted of four transgenic lines, a wild type and a local check. There were six plants of each line per plot replicated three times. The experimental trial size was 41 × 25.5 m. The spacing of the plants was 1.5 m (between rows) by 1 m (within rows). In total, each replication had 36 cassava plants giving a total of 108 plants in all the three replications. The trial had two border rows of wild type cassava around the trial.

### Sample collection, preservation and transportation

Transgenic cassava leaves and roots were sampled from the field during harvesting at 12 Months after Planting (MAP). The roots were gently harvested from soil that was dampened a day before by gently scraping the soil away with a forked *jembe* before using a knife to loosen the soil from the roots. All the roots were removed from the stem and three largest root sampled from each plant and labeled A (largest root), B (medium) and C (smallest of the three). The roots were cleaned, air-dried then waxed (retain β-carotene), finally packaged in three tiers (three layers of packaging) and well labeled. Waxing was done by heating candles to 60 °C in a pan and allowed to cool off to 55 °C before dipping the whole root and placing it on tray to dry off for 2 min then packaged in three tiers. This was done under umbrella shade and tents limiting exposure to sunlight to prevent β-carotene degradation. The leaves were picked from one plant in every plot randomly sampled. Four leaf samples were taken from the apical leaves and kept in a zipped polythene bag and preserved in dry ice and packaged in three tiers. The samples were transported to the KARI-NARL Biotechnology Centre Biosafety Level II lab via air under supervision of a Kenya Plant Health Inspectorate Service (KEPHIS) Inspector.

### Sample preparation

The outer cover of cassava was peeled and cortex chopped into 1 cm^3^ cubes from the proximal, central and distal ends of all the cassava root samples under coloured bulb light. They were mixed to make a composite and packaged in a flat bottomed 50 ml falcon tube then their fresh weight taken. The samples were frozen in liquid Nitrogen and kept in −20 °C freezer to prevent thawing. The tube was covered with aluminum foil (prevent β-carotene degradation). The samples were later transferred to −80 °C freezer awaiting lyophilisation.

### Lyophilisation

The root samples were freeze dried using the Edward Modulyo Freeze dryer for 72 h at −50 °C and 10^−1^ atm. The samples were ground using mortar and pestle and transferred to sterile labeled 14 ml falcon tube and covered with aluminum foil. The leaf samples were also freeze dried for 24 h and ground to fine powder using mortars and pestle and kept in sterile labeled 14 ml falcon tube and covered with aluminum foil. The samples were kept in −80 °C freezer until use.

### Assaying of transgenic lines by RT-PCR

#### RNA isolation

RNA was isolated from 0.1 g of transgenic roots flour and 0.02 g leaf powder using modified Dellaporta protocol (1983). The RNA was quantified using NanoDrop 2,000 from KEPHIS and ranged between 800 and 1,200 ng/µl. The concentration was standardized to 50 ng/µl for use in RT-PCR.

#### Reverse transcriptase PCR (RT-PCR)

The following primers were obtained from Donald Danforth Plant Science Centre (DDPSC):

1. DXS (583 bp)
2. *crtB* (409 bp)
3. *npt*II (406 bp)

Positive control was obtained from DDPSC. RNase free water was used as negative control.

One step RT-PCR kit (Qiagen) was used to amplify the above mentioned genes as described in Table 1.

**Table 1:** RT-PCR conditions for amplification of DXS and *crtB* genes

Step	Time	Temp (°C)
Reverse transcription	30 min	55
Initial PCR activation	5 min	95
35 cycles		
Denaturation	30 s	95
Annealing–(DXS*/crtB)*	30 s	51
Extension	45 s	72
Final extension	10 min	72
Final hold	∞	4

#### Visualization

The RT-PCR products were ran on 1.5 % agarose gel electrophoresis using the MacroDrivel powerpack at 150 V for 90 min using 0.5X TBE buffer. GeneRuler ladder −100 bp was included as reference.

The size of bands was visualized under the UV Transilluminator (GelVe–GVM 2530) and gel photos taken for analysis.

### Statistical analysis

Molecular analysis was achieved through observing the size of bands visualized under the UV Transilluminator and gel photos taken. Scoring was done for presence or absence of the target bands DXS–583 bp, *crtB*–409 bp and *npt*II–406.

## Results

### Evaluation of β-carotene genes in transgenic cassava roots

**Fig. 1:**
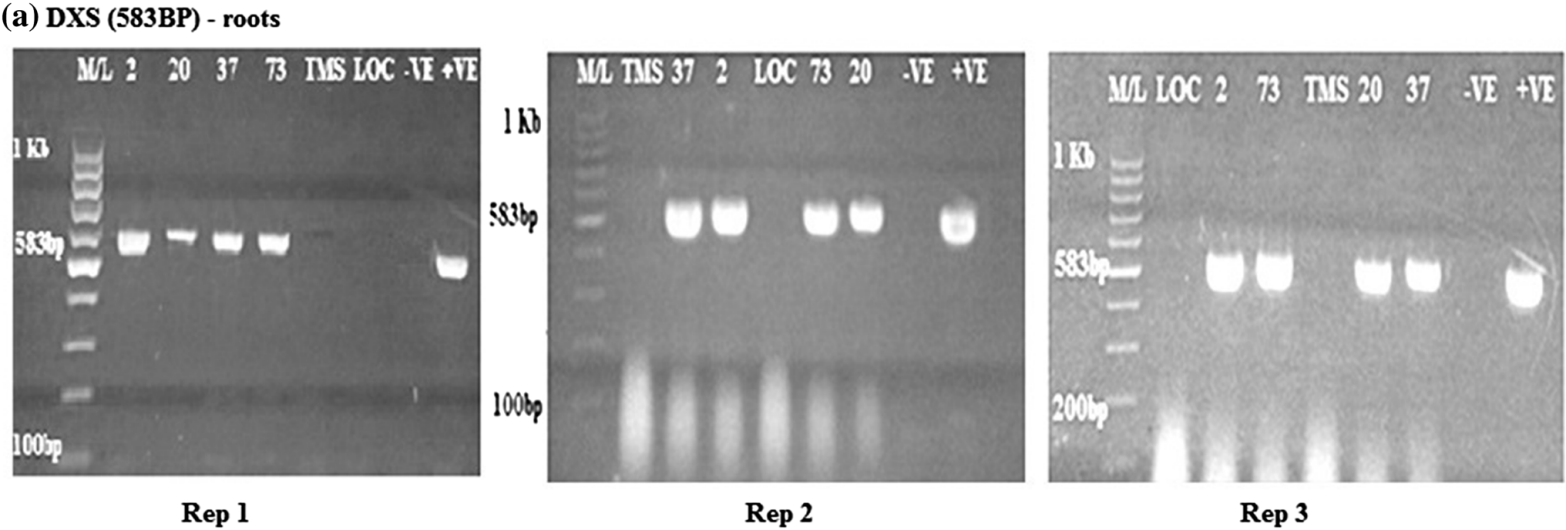
RT-PCR amplification of the DXS gene in transgenic roots obtained from rep 1, 2 and 3, respectively, showing the *positive* and *negative lines* for the DXS gene. M/L: Molecular ladder 100 bp (GeneRuler); 2, 20, 37 and 73: Transgenic lines, TMS: TMS 60444 non-transgenic cultivar; LOC: Local check (*yellow fleshed*); −VE–*Negative control*; +VE–*Positive control*

**Fig. 2:**
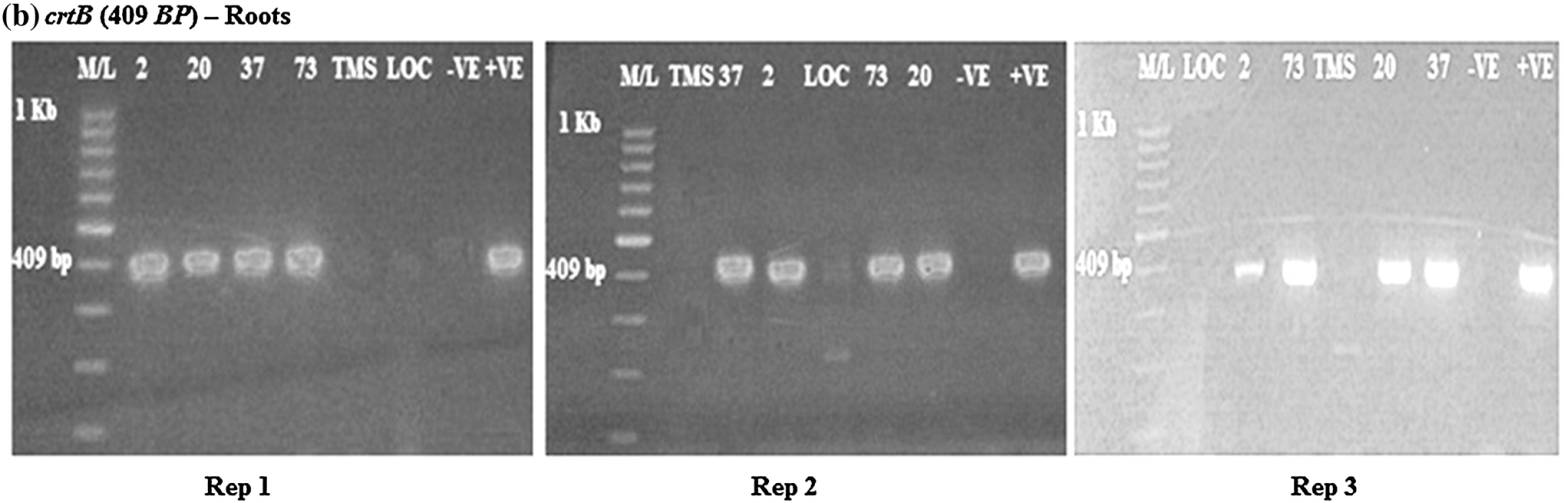
RT-PCR amplification of the *crtB* gene in transgenic roots in rep 1, 2 and 3, respectively, showing the* positive* and* negative lines *for the *crtB* gene. M/L: Molecular ladder 100 bp (GeneRuler); 2, 20, 37 and 73: Transgenic lines, TMS: TMS 60444 non-transgenic cultivar; LOC: Local check (*yellow fleshed*); −VE–*Negative control*; +VE–*Positive control*

**Fig. 3:**
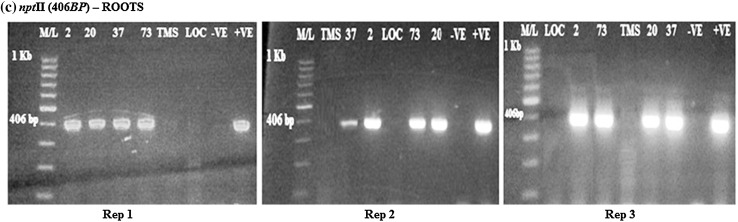
RT-PCR amplification of the *npt*II gene in transgenic roots in rep 1, 2, and 3, respectively, showing* positive* and* negative lines* for the *npt*II gene. M/L: Molecular ladder 100 bp (GeneRuler); 2, 20, 37 and 73: Transgenic lines, TMS: TMS 60444 non-transgenic cultivar; LOC: Local check (*yellow fleshed*); −VE–*Negative control*; +VE–*Positive control*

Amplification of the DXS*, crtB* (β-carotene gene) and *npt*II genes on the roots and products was compared to the size of the specific gene of interest. The DXS gene (583 bp) was present and expressed in the transgenic cassava roots (Fig. 1). All the transgenic lines tested positive for expression of the DXS genes. The wild type TMS 60444 did not have band of interest and this was the case with the local check *Nyaboda*. Amplicons were also present for the *crtB* (409 bp) and *npt*II (406 bp) genes in the cassava transgenic roots lines 2, 20, 37 and 73 as shown in Figs. 2 and 3. The wild type did not show any band. The amplification was done for the six samples in each of the three reps. The local check did not have band of interest in all the three genes amplified. All the transgenic cassava roots were positive for the DXS*, crtB* and *npt*II genes. This characteristic was repeated in all the three replications for conformity. The wild type and local check were negative of the three genes.

### Evaluation of β-carotene gene in transgenic cassava leaves

**Fig. 4:**
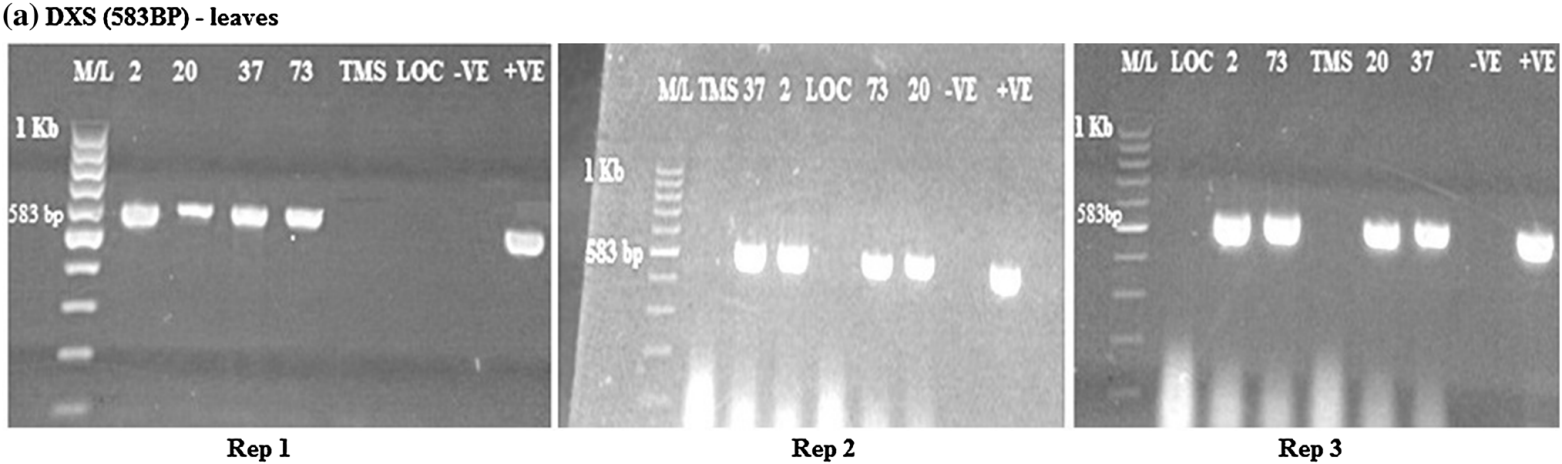
RT-PCR amplification of the DXS gene in transgenic cassava leaves in samples from rep 1, 2 and 3, respectively, showing* positive* and* negative lines* for DXS gene. M/L: Molecular ladder 100 bp (GeneRuler); 2, 20, 37 and 73: Transgenic lines, TMS: TMS 60444 non-transgenic cultivar; LOC: Local check (*yellow fleshed*); −VE–*Negative control*; +VE–*Positive control*

**Fig. 5:**
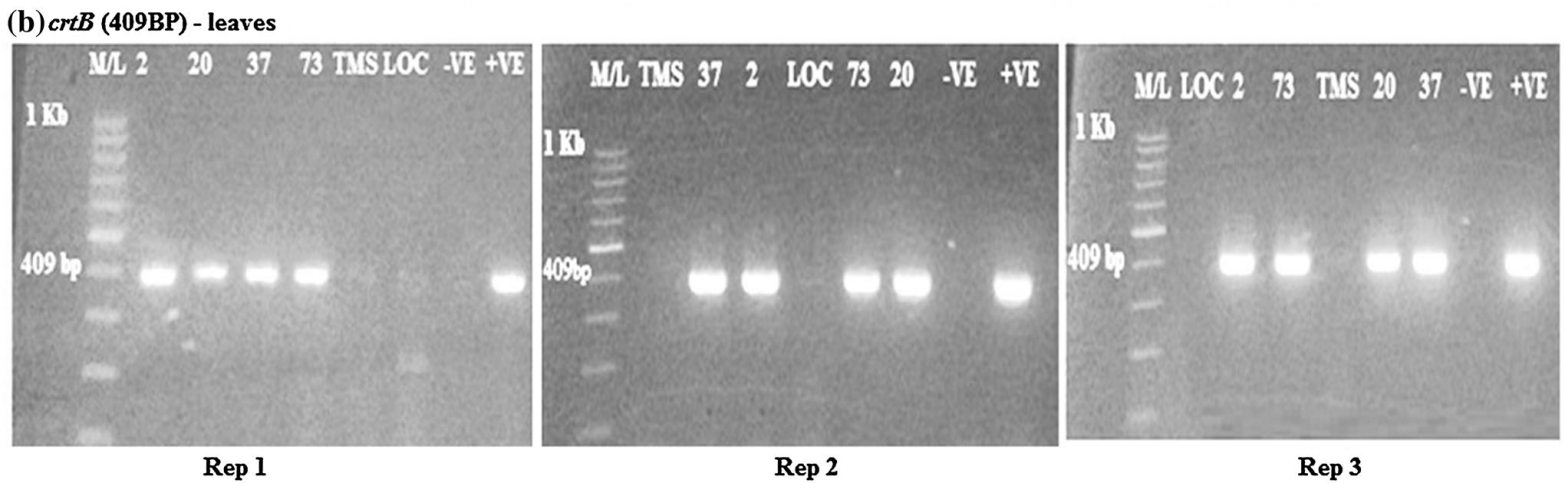
RT-PCR amplification of the *crtB* gene in transgenic cassava leaves samples from rep 1, 2 and 3, respectively, showing *positive* and *negative lines* for *crtB* gene. M/L: molecular ladder 100 bp (GeneRuler); 2, 20, 37 and 73: Transgenic lines, MS: TMS 60444 non-transgenic cultivar; LOC: Local check (*yellow fleshed*); −VE–*Negative control*; +VE–*Positive control*

**Fig. 6:**
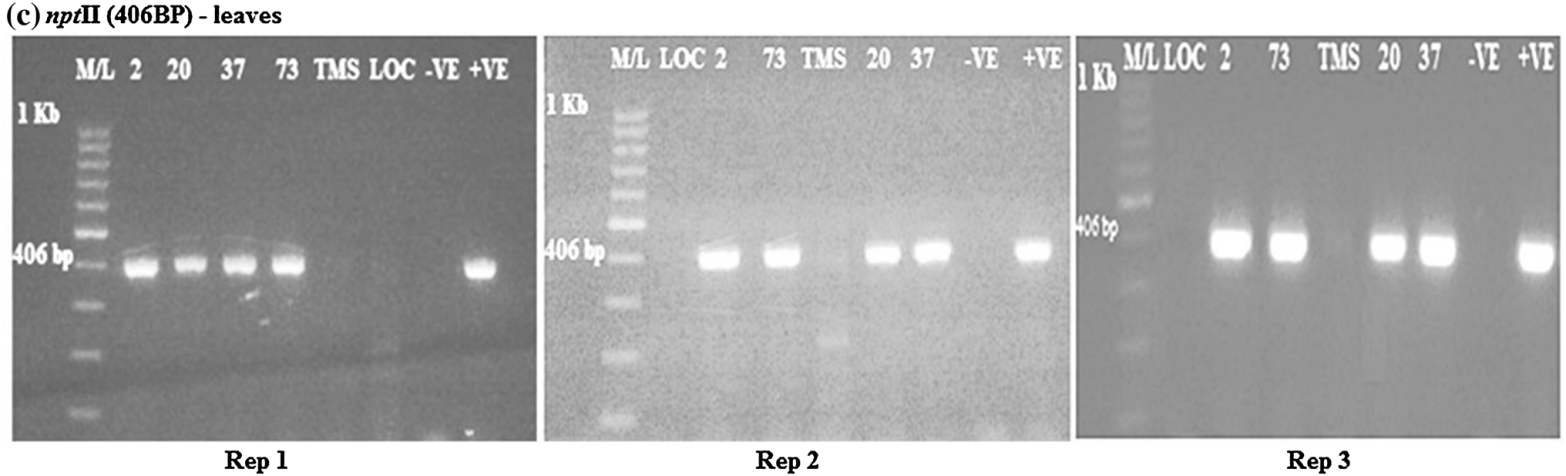
RT-PCR amplification of the *npt*II gene in transgenic cassava leaves samples from rep 1, 2 and 3, respectively, showing *positive* and *negative lines* for *npt*II gene. M/L: Molecular ladder 100 bp (GeneRuler); 2, 20, 37 and 73: Transgenic lines, TMS: TMS 60444 non-transgenic cultivar; LOC: Local check (*yellow fleshed*); −VE–*Negative control*; +VE–*Positive control*

From the analysis, DXS, *crtB* and *npt*II genes were amplified in the transgenic cassava leaves as bands were present. DXS was amplified at 583 bp, *crtB* at 409 bp and *npt*II at 406 bp (Figs. 4, 5, 6). The amplified DXS, *crtB* and *npt*II genes were not observed in the wild type and local check. There were three replications in all the cases.

From the gel photos, it was confirmed that the DXS and *crtB* genes were expressed in the leaves for all the transgenic cassava. The results were negative for the wild type and local variety. All the transgenic cassava lines were positive for *npt*II gene in both the leaves and roots.

## Discussion

From the data presented, it is evident that the promoter used, patatin drives expression of the DXS, *crtB* and *npt*II gene in the roots of transgenic cassava as expected. The bands of interest were present in all the transgenic cassava roots and leaves for the DXS, *crtB* and *npt*II genes. Their product sizes (in bp) were also comparable to the respective genes DXS–583 bp, *crtB*–409 bp and *npt*II–406 bp. This indicates that the genes were present and expressed in the cassava roots of the transgenic lines. The Class I Patatin promoter is a strong tuber specific promoter. It is usually activated during tuber formation in potato and transgenic cassava (Jefferson et al. 1990; Zain 2010). However, from RT-PCR analysis, one cannot tell the level of expression unless analysis of the final product accumulation (β-carotene) is quantified using different molecular analysis methods such as quantitative PCR or real time PCR.

DXS, *crtB* and *npt*II genes were present and expressed in the leaves. All the transgenic cassava leaves tested positive for the three genes despite the use of the Patatin promoter. The wild type tested negative for the three genes. This suggests that there was leakage of the transgenes. The wild type tested negative for the three genes. It has been found out that at times, the promoter drives low level of gene expression in other tissues such as leaves, roots and stem in potato under conditions that stimulate the need for the accumulation of starch in storage organs such as high levels of sucrose (Mario et al. 1989). In addition, reverse transcriptase PCR on the roots cannot quantify the level of expression. (Rotcha-Sosa et al. 1989) noted that a number of environmental, metabolic and developmental factors contributed to patatin gene expression. From the CFT it is evident that one or more of developmental or environmental conditions played a role in inducing the expression of the promoter gene in the leaves though this was not confirmed in the current study. Studies on amino acid inducing Class I patatin promoter in Potato have shown that amino acid Proline is a potent inducer in Arabidopsis (Hellmann et al. 2000; Nielsen et al. 1998). Moreover, Wenzler et al. 1989 proved that sucrose also plays a role in inducing patatin gene expression in the transgenic tobacco leaves. These inducers or others not known could have played a role in the expression of the Patatin in leaves but studies on the same in transgenic cassava are not in public domain yet. Further nutritional analysis on the leaves for the transgenic cassava grown in Kenyan environment needs to be done. This will help unravel the uncertainty of whether the expression of this gene in the leaves could have any effect on other nutrients rich in leaves such as protein, toxins such as cyanide and other metabolic pathways.

## Abbreviations

DXS: 1 Deoxy-d-xylulose-5-phosphate synthase gene
*crtB*: Bacterial version of phytoene synthase
*npt*II: Neomycin phosphotransferase gene
CFT: Confined field trial
RT-PCR: Reverse transcriptase polymerase chain reaction

## References

[CR1] Alison G, Rachel A, Busie M, Dixon CE, Sally M, Rhoda N, Gichuki S, Ada M, Manary J (2010). Children consuming cassava as a staple food are at risk for inadequate zinc, iron, and Vitamin A intake. Plant Foods Hum Nutr.

[CR01] Angela M (2007) Case study 3–7. In: Pinstrup-Andersen P, Cheng F (eds) Biofortification as a Vitamin A deficiency intervention in Kenya, Food policy for developing countries: case studies. http://cip.cornell.edu/dns.gfs/1200428157

[CR4] Centre D (2013) Biocassava plus participants. Scientists and research. http://www.danforthcenter.org/scientists-research/research-institutes/institute-for-international-crop-improvement/crop-improvement-projects/biocassava-plus/participants

[CR5] Dellaporta SL, Wood J, Hicks JB (1983). A plant DNA miniprepration: version II. Plant Mol Biol Rep.

[CR6] Fregene M, Sayre R, Fauquet C, Anderson P, Taylor N (2010) Opportunities for biofortification of cassava for sub-Saharan Africa: the biocassava plus program. Promoting Health by Linking Agriculture, Food, and Nutrition. NABC Report 22:81–90

[CR7] HARVESTPLUS (2008) Target crops: CASSAVA available online at: http://www.harvestplus.org. Accessed 10 Jul 2013

[CR8] Hellmann H, Funck D, Rentsch D, Frommer WB (2000). Hypersensitivity of an arabidopsis sugar signalling mutant toward exogenous proline application. Plant Physiol.

[CR9] Horna D, Melinda S, Jose F (2006). Assessing the potential economic impact of genetically modified crops in Ghana: tomato, garden egg.

[CR10] Jaetzold R, Schmidt H, Hornetz B, Shisanya CA (2009) Farm management handbook of Kenya. Natural conditions and farm information, vol 11/C, 2nd edn. Ministry of Agriculture/GTZ, Nairobi, Kenya

[CR11] Jefferson R, Goldsbrough A, Bevan M (1990). Transcriptional regulation of a Patatin-1 gene in potato. Plant Mol Biol.

[CR12] Kariuki CW, Kamau JW, Mbwika J, Munga TL, Makhoha AO, Wambugu S, Tunje T, Nzioki S (2002) A report on cassava sub-sector analysis for Kenya

[CR19] Kimathi M, Ngeli P, Wanjiru J (2007) Final report: analyzing value chains for specific commodities: the case of Cassava flour. ECAPACA report, pp 17–35

[CR13] Kiura JN, Mutegi CK, Kibet P, Danda MK (2005) Cassava production, utilisation and marketing in coastal Kenya. A report of a survey on cassava enterprise conducted between July and October 2003 in Kwale, Kilifi, Mombasa and Malindi districts. Internal Report No. 35, KARI-Mtwapa

[CR14] Lancaster PA, Brooks JE (1983). Cassava leaves as human food. Econ Bot.

[CR15] Manyong VM, Bamire AS, Sanusi IO, Awotide DO (2004) Ex-Ante evaluation of nutrition and health benefits of biofortified cassava roots in Nigeria: the Dalys approach. In: shaping the future of african agriculture for development: the role of social scientists. Proceedings of the inaugural symposium, Dec 2004, Nairobi, Kenya. African Association of Agricultural Economists, pp 1–8

[CR16] Mario R, Uwe S, Wolf F, Marina S, Jeff S, Lothar W (1989). Both developmental and metabolic signals activate the promoter of a class I patatin gene. EMBO J.

[CR17] Martin T, Hellmann H, Schmidt R, Willmitzer L, Frommer WB (1997). Identification of mutants in metabolically regulated gene expression. Plant J.

[CR18] Ministry of Agricultre, Kenya (2007). National Policy on cassava Indutry. Naumkina E. M., Yu. P. Bolyakina, and G. A. Romanov (2007). Organ-specificity and inducibility of patatin Class I promoter from potato in transgenic arabidopsis plants. Russ J Plant Physiol.

[CR2] Mwaniki A (2007) Case study #3–7, Biofortification as a Vitamin A deficiency intervention in Kenya. In: Pinstrup-Andersen P, Cheng F (eds) Food policy for developing countries: case studies, p 11. http://cip.cornell.edu/dns.gfs/1200428157

[CR20] Naumkina EM, Bolyakina YuP, Romanov GA (2007). Organ-specificity and inducibility of patatin Class I promoter from potato in transgenic arabidopsis plants. Russ J Plant Physiol.

[CR21] Nielsen TH, Krapp A, Roeper-Schwarz U, Stitt M (1998). The sugar-mediated regulation of genes encoding the small subunit of rubisco and the regulatory subunit of ADP glucose pyrophosphorylase is modified by phosphate and nitrogen. Plant Cell Environ.

[CR23] Obiero HM, Whyte JAB, Legg JP, Akhwale MS, Maling’a J, Magut T (2007) Successful restoration of cassava production in Western Kenya. In: Proceedings of the 13th ISTRC Symposium, Tanzania, pp 682–685

[CR3] Ospina B, Segovia R, Bedoya A (2007) Micropropagation of cassava plants through the temporary immersion system and hardening of massive numbers of cassava vitroplants. In: CIAT Conference proceedings. pp 161–173

[CR24] Paine A, Catherine A, Sunandha C, Rhian M, Mike J, Gareth V, Susan Y, Edward H, Jessica L, Aron L, Rachel D (2005). Improving the nutritional value of golden rice through increased pro-vitamin A content. Nat Biotechnol.

[CR25] Rotcha-Sosa M, Sonnewald U, Frommer W, Stratmann M, Schell J, Willmitzer L (1989). Both developmental and metabolic signals activate the promoter of a Class I patatin gene. EMBO J.

[CR26] Shewmaker CK, Sheehy JA, Daley M, Colburn S, Ke DY (1999). Seed-specific overexpression of phytoene synthase: increase in carotenoids and other metabolic effects. Plant J.

[CR27] UNCST (2009) A handbook for the conduct of confined field trials of transgenic cassava in uganda. In: Compliance with the standard operating procedures for conducting confined field trials. UNCST CFT Publication series

[CR28] Wenzler H, Mignery G, Fisher L, Park W (1989). Sucrose-regulated expression of a chimeric Potato tuber gene in leaves of transgenic tobacco plants. Plant Mol Biol.

[CR22] Norhidayu Muhamad Zain (2010). Enhancement of provitamin a in potato tuber using overexpression and silencing of carotenogenic genes under control of promoter. Basic Biotechnology.

